# Novel surface features for automated detection of focal cortical dysplasias in paediatric epilepsy

**DOI:** 10.1016/j.nicl.2016.12.030

**Published:** 2016-12-30

**Authors:** Sophie Adler, Konrad Wagstyl, Roxana Gunny, Lisa Ronan, David Carmichael, J Helen Cross, Paul C. Fletcher, Torsten Baldeweg

**Affiliations:** a Developmental Neurosciences, UCL Great Ormond Street Institute of Child Health, University College London, London, UK; b Great Ormond Street Hospital for Children, London, UK; c Brain Mapping Unit, Institute of Psychiatry, University of Cambridge, UK; d Cambridge & Peterborough NHS Foundation Trust, Cambridgeshire, UK

**Keywords:** AUC, area under the curve, FCD, focal cortical dysplasia, FLAIR, fluid-attenuated inversion recovery, LCD, local cortical deformation, LGI, local gyrification index, PCA, principal component analysis, ROC, receiver operator characteristic, FCD, Intractable epilepsy, Structural MRI, Automated classification, Paediatric

## Abstract

Focal cortical dysplasia is a congenital abnormality of cortical development and the leading cause of surgically remediable drug-resistant epilepsy in children. Post-surgical outcome is improved by presurgical lesion detection on structural MRI. Automated computational techniques have improved detection of focal cortical dysplasias in adults but have not yet been effective when applied to developing brains. There is therefore a need to develop reliable and sensitive methods to address the particular challenges of a paediatric cohort.

We developed a classifier using surface-based features to identify focal abnormalities of cortical development in a paediatric cohort. In addition to established measures, such as cortical thickness, grey-white matter blurring, FLAIR signal intensity, sulcal depth and curvature, our novel features included complementary metrics of surface morphology such as local cortical deformation as well as post-processing methods such as the “doughnut” method - which quantifies local variability in cortical morphometry/MRI signal intensity, and per-vertex interhemispheric asymmetry. A neural network classifier was trained using data from 22 patients with focal epilepsy (mean age = 12.1 ± 3.9, 9 females), after intra- and inter-subject normalisation using a population of 28 healthy controls (mean age = 14.6 ± 3.1, 11 females). Leave-one-out cross-validation was used to quantify classifier sensitivity using established features and the combination of established and novel features.

Focal cortical dysplasias in our paediatric cohort were correctly identified with a higher sensitivity (73%) when novel features, based on our approach for detecting local cortical changes, were included, when compared to the sensitivity using only established features (59%). These methods may be applicable to aiding identification of subtle lesions in medication-resistant paediatric epilepsy as well as to the structural analysis of both healthy and abnormal cortical development.

## Highlights

• Automated classifier to identify focal cortical dysplasias in a paediatric cohort
• Novel measure of morphology – local cortical deformation
• “Doughnut method” to quantify local changes in cortical structure
• Interhemispheric, homotopic comparison of surface-based measures
• Classifier sensitivity improved from 53% to 73% with incorporation of novel features

## 1 Introduction

Focal cortical dysplasias (FCDs) are the most common cause of surgically remediable drug-resistant epilepsy in children (Lerner et al., 2009). Surgical resection can result in reduced need for anti-epileptic medication, reduced frequency or most commonly complete absence of seizures (Cross, 2002, D'Argenzio et al., 2011, D'Argenzio et al., 2012) There is evidence too that it can even improve developmental outcome (Skirrow et al., 2011, Skirrow et al., 2015). The challenge in many cases is to accurately locate the area of responsible tissue. Surgical outcome is significantly improved when lesions are identified on MRI scans pre-surgically (Téllez-Zenteno et al., 2010). However between 50 and 80% of FCDs are too subtle to detect by conventional radiological analysis of MRI scans (Besson et al., 2008). While progress has been made in improving the detection of FCDs in adults using structural neuroimaging techniques (Thesen et al., 2011, Wang et al., 2015) and automated classifiers (Ahmed et al., 2015, Hong et al., 2014), automated lesion classification has not been attempted in a solely paediatric cohort despite this being a congenital condition (Chen et al., 2014). Therefore an automated tool capable of improving the detection of FCD in the paediatric population would represent an important step in improving the quality and consistency of presurgical evaluation with implications for surgical outcome.

Applying automated lesion detection methods in a paediatric population raises a number of unique challenges. First, between the ages of one and 18 the cortex undergoes major structural changes including cortical thickening and thinning (Giedd et al., 2015, Gogtay et al., 2004, Raznahan et al., 2011, Shaw et al., 2008), as well as changes in gyrification (Li et al., 2014) and myelination (Deoni et al., 2015, Whitaker et al., 2016a, Whitaker et al., 2016b), thus identifying focal abnormalities in cortical structure requires careful consideration of developmental trajectories. For example, an apparent thickening of cortex may not necessarily signify an abnormality for a given individual at a given age. Second, motion artefacts are more prevalent in paediatric imaging affecting the accuracy of established surface-based features (Ducharme et al., 2015). Sensitivity to detect FCDs may therefore be improved by novel features and post processing methods measuring different aspects of cortical structure.

FCDs include a spectrum of localized malformations of cortical development, manifesting as an array of characteristic radiological features. One classification system developed by the International League Against Epilepsy (ILAE) (Blümcke et al., 2010) defines histological subtypes as follows. FCD type I have abnormal radial and tangential lamination; FCD type II are associated with aberrant cytology, such as large dysmorphic neurons plus/minus balloon cells; and FCD type III occurs with another lesion, e.g. hippocampal sclerosis. Radiologically, FCDs have been associated, albeit inconsistently, with a range of features including local cortical thinning or thickening, blurring of the grey-white matter boundary, abnormal cortical folding patterns, increased signal intensity on FLAIR/T2-weighted MRI (including the transmantle sign in FCD Type IIB) and interhemispheric asymmetry in any of the above traits (Colombo et al., 2003, Colombo et al., 2012, Yagishita et al., 1997). The variable presentation of these radiological features and the fact that they are often small and subtle, means that they are easily missed on visual inspection by radiologists (Wagner et al., 2011).

To overcome the difficulty of radiological assessment of FCDs, automatic detection methods build a series of morphological measures into an identification algorithm to improve detection rate (Ahmed et al., 2015, Besson et al., 2008, Hong et al., 2014, Thesen et al., 2011). For example, surface-based techniques may be used to calculate various measures such as cortical thickness (Fischl and Dale, 2000), signal intensity in the grey or white matter (Salat et al., 2009), local gyrification index (LGI) (Schaer et al., 2008), sulcal depth and curvature (Fischl et al., 2004) at each point on the cortical surface (henceforth vertices). These measures provide an improved detection rate, with rates as high as 74% in adult cohorts (Hong et al., 2014), compared to other approaches such as diffusion tensor imaging (DTI), voxel-based morphometry (VBM), (see reviews: (Bernasconi et al., 2011, Martin et al., 2015)). However automated classification using surface-based measures has not been applied to a paediatric cohort, and, owing to the particular differences between adult and paediatric brains it is unclear that current approaches are suitable or would yield similar results.

Our overall approach to develop a tool for automated FCD detection, which addresses the particular challenges of a paediatric cohort, was to optimize the ability to find and quantify each area of cortex in terms of how it differed from healthy cortex. To this end, we calculated structural measures and applied post-processing methods to quantify a number of radiological identifiers of focal cortical dysplasias. First, established structural markers of FCD - cortical thickness, intensity contrast at the grey-white matter boundary and FLAIR signal intensity - have normal developmental and regional differences which can obscure locally abnormal values within an FCD. To address this we normalised measures within subjects, calculated interhemispheric asymmetries of these measures and normalised the values for each vertex relative to a group of healthy paediatric controls. Moreover, FCDs are characterised by focal changes in these structural markers and thus subtle lesions should be identifiable as local areas of abnormal cortical thickness, grey-white matter contrast and FLAIR signal intensities. We quantified these local changes by creating a “doughnut” method, which calculates the difference between an area of cortex and its surrounding annulus at each vertex, highlighting where these differences are greatest. Finally noise and particularly motion artefacts are common problems in paediatric scans. Intrinsic curvature, a small scale measure of cortical shape deformation, only requires an accurate pial surface and is unaffected by motion-related inaccuracies in the segmentation of the grey-white matter boundary. Furthermore, it is more sensitive to subtle cortical abnormalities than larger scale folding parameters measures such as LGI (Ronan et al., 2014). We therefore developed a measure of local cortical deformation (LCD) based on the magnitude of intrinsic curvature surrounding each vertex (Ronan et al., 2011), as a more robust measure of cortical shape. The added value of these structural markers and post-processing methods – local cortical deformation, interhemispheric asymmetry and the “doughnuts” of structural measures - were then combined with the established surface-based metrics for FCD detection (cortical thickness, grey-white matter intensity contrast, FLAIR signal intensity, curvature and sulcal depth) in a neural network trained to classify cortical regions into lesional and nonlesional vertices. Furthermore we directly compared our measure of cortical shape, LCD, with the existing measure LGI.

## 2 Materials and methods

### 2.1 Participants

A retrospective cohort of 27 patients with radiologically defined FCD (mean age = 11.57 ± 3.96, range = 3.79–16.21 years, 10 females) who underwent 3D T1 and FLAIR imaging on the 1.5T MRI scanner at Great Ormond Street Hospital as part of their clinical workup were studied, following permission by the hospital ethical review board. Cases were identified by searching the medical reports for a radiological diagnosis of FCD. Exclusion criteria were patients scanned using a different MRI scanner or protocol. The following information from the medical notes was gathered for all patients included in this study: age at epilepsy onset, duration of epilepsy, radiological report, current anticonvulsant medications and, where applicable, post-surgical histology. A control group of 28 term-born children with no history of any neurological diagnosis (mean age = 14.57 ± 3.06, range = 10.1–19.75 years, 11 females) were recruited by advertisement.

### 2.2 MR imaging

All participants were scanned on a 1.5T Avanto MRI scanner (Siemens, Elangen, Germany). Three-dimensional data sets were acquired using a T_1_-weighted 3D-FLASH sequence (TR = 11 ms, TE = 4.94 ms, FOV = 256 × 256 mm, flip angle = 15°, voxel size = 1 × 1 × 1 mm^3^) and T_2_-weighted FLAIR sequence (TR = 6000 ms, TE = 353 ms, TI = 2200 ms, FOV = 256 × 256 mm, flip angle = 15°, voxel size = 1 × 1 × 1 mm^3^). Anonymised FLAIR and T1 volumetric scans were rated from one to five according to severity of motion artefact. The following classification system was used: 1) no visible motion artefacts, 2) subtle artefacts visible, 3) mild ringing artefacts, 4) severe ringing artefacts and 5) adjacent gyri indistinguishable due to motion.

### 2.3 Cortical reconstruction

*FreeSurfer* software v5.3 (Dale, 1999, Fischl and Dale, 2000, Fischl et al., 1999) was used to generate the cortical reconstructions and to co-register the FLAIR scans to T1-weighted images. In outline, *FreeSurfer* firstly sub-samples the raw image data voxels to 1mm^3^ isotropic voxels. The data is then normalised for intensity and RF-bias field inhomogeneities are modeled and removed. The skull is then removed from all of the images using a skull-stripping algorithm (Ségonne et al., 2004). Subsequently, cerebral white matter is identified, and the hemispheres are separated, tessellated and deformed to create accurate smooth mesh representations of the grey-white matter interface and pial surface, with approximately 150,000 vertices per hemisphere. Within-subject registration of FLAIR scans to T1 images was performed using a boundary-based cost function; the white-matter boundary is mapped to the FLAIR image and the FLAIR intensity is sampled per-vertex either side of the boundary. The difference in intensity between each pair of intensities is then used to calculate the cost function. All of the reconstructions were checked and any inaccuracies were manually corrected. Five participants were excluded due to severe motion artefacts. There was no significant difference in age between the included and excluded participants (Mann-Whitney U: − 1.53, p = 0.13). However, within the included patients, younger patients tended to have higher motion artefact ratings (Spearman's rho = − 0.36, p = 0.10).

### 2.4 Lesion masks

Manual lesion masks were created for the 22 participants, on axial slices of the volumetric scan. Lesions were identified combining information from T1 and FLAIR images, previous radiological reports, reports from multi-disciplinary team meetings as well as oversight from a consultant paediatric neuroradiologist. The lesion masks were then registered onto the cortical surface reconstructions.

### 2.5 Measures of morphological/intensity features

*FreeSurfer* was used to calculate the established measures: cortical thickness, grey-white matter intensity contrast, curvature, sulcal depth and FLAIR intensity at each vertex of the 3D cortical reconstruction. Thickness was calculated as the mean minimum distance between each vertex on the pial and white matter surfaces, generating a millimeter-scale measure of the thickness of the cortex. Further details of these methods are available in (Fischl and Dale, 2000). Grey-white matter intensity contrast was calculated as the ratio of the grey matter signal intensity to the white matter signal intensity (Salat et al., 2009). The grey matter signal intensity was sampled at a distance of 30% of the cortical thickness above the grey-white matter boundary. The white matter signal intensity was sampled 1 mm below the grey-white matter boundary. Lesions with blurring of the grey-white matter boundary are expected to have low grey-white matter intensity contrast values compared to healthy cortex. FLAIR intensity was sampled at the grey-white matter boundary as well as at 25%, 50% and 75% depths of the cortical thickness and at − 0.5 mm and − 1 mm below the grey-white matter boundary. Mean curvature was measured at the grey-white matter boundary as 1/r, where r is the radius of an inscribed circle and is equal to the mean of the principal curvatures *k*_1_ and *k*_2_ (Pienaar et al., 2008)_._ The dot product of the movement vector of the cortical surface during inflation is used to calculate the sulcal depth. Shallow, gyral areas of the brain move inwards during inflation and have a negative value whereas, deep, sulcal areas move outwards and have a positive value.

### 2.6 “Doughnut” method

**Fig. 1:**
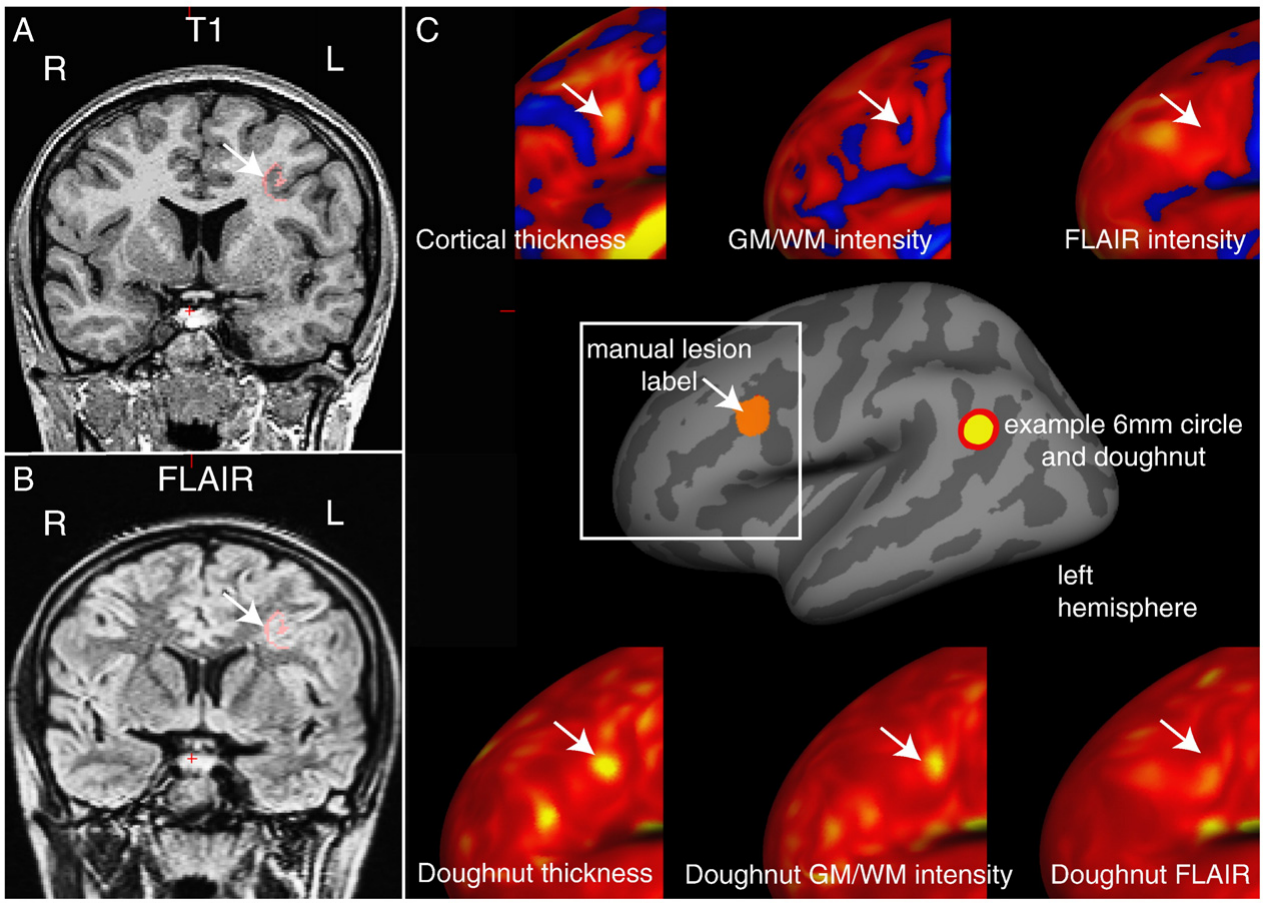
Example of “doughnut” method maps in a patient with a left middle frontal sulcus FCD. A) T1 image B) FLAIR image - manual lesion label in pink, white arrow indicates lesion. C) Inflated surface view with manual lesion label (orange) and example of 6 mm doughnut and circle. Upper panel – intra-subject normalised cortical thickness, grey-white matter contrast and FLAIR intensity (sampled at 50% cortical thickness) overlays around lesion area (white square). Lower panel – “doughnut” thickness, “doughnut” grey-white matter intensity and “doughnut” FLAIR (sampled at 50% cortical thickness). This lesion is characterised by a subtle increase in cortical thickness, though much less thick than the insula (bright yellow), subtle decrease in contrast at the grey-white matter boundary and no obvious FLAIR hyperintensity. “doughnut” thickness and “doughnut” grey-white matter intensity contrast highlight lesion, in this particular example “doughnut” FLAIR is of less use. All surface measures also identify other areas of cortex with extreme values and must therefore be used in combination.

A 6 mm radius circle was centred on a vertex on the inflated surface (Fig. 1). A surrounding “doughnut” of cortex of the same area (~ 113 mm^2^) was placed around it. The cortical thickness, grey-white matter intensity contrast or FLAIR signal intensity was measured within the circle and within the doughnut. A *t*-test was used to compare the thickness/grey-white matter intensity contrast in the circle and doughnut. This measurement was repeated per vertex over the inflated surface. “Doughnut” thickness, “doughnut” intensity contrast and six “doughnut” FLAIR signal intensity maps were created per participant using the log of the per-vertex, *t*-test p-values. “Doughnut” maps were smoothed using a 10 mm FWHM Gaussian kernel, to remove noise while maintaining local specificity. A 6 mm radius was used as it offered a balance between identifying local changes in thickness/intensity on a scale finer than gyral/sulcal changes, and insensitivity to motion artefact, a common problem when analysing paediatric MRI data (Reuter et al., 2015). The code is available from https://github.com/kwagstyl/FCDdetection/.

### 2.7 Local cortical deformation

**Fig. 2:**
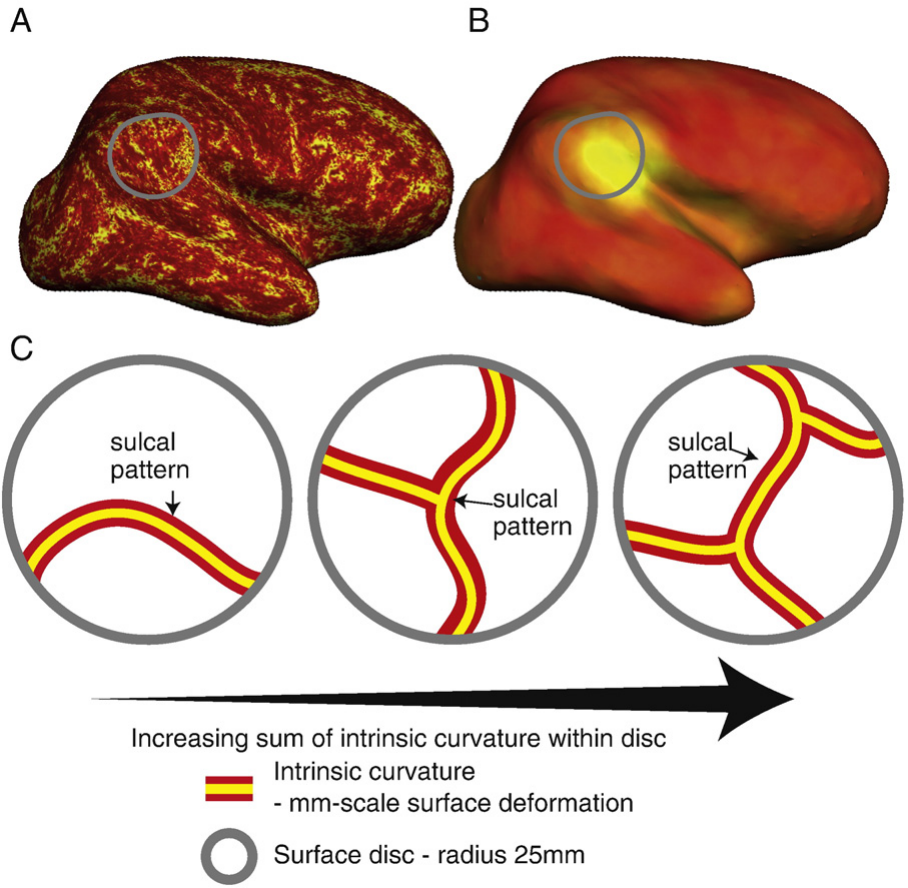
Local cortical deformation. A) Surface overlay of per-vertex intrinsic curvature. The modulus of intrinsic curvature is summed within a 25 mm disc (grey circle) to calculate per-vertex local cortical deformation (B). C) Local cortical deformation is increased either due to increased sulci fundi (i.e. more folds) or small-scale surface deformation.

Cortical deformation, also known as intrinsic or Gaussian curvature, was calculated at a mm scale across the pial surface (Ronan et al., 2011). As the product of the principal curvatures, *k*_1_ and *k*_2_, it is extremely sensitive to local surface deformations, and particularly high in sulcal fundi. A 25 mm radius ring was centred on a vertex and the sum of the intrinsic curvature within the ring was computed (Fig. 2). This process was repeated per vertex across the cortical surface to create a measure of local cortical deformation. A 25 mm ring was chosen as in normal folded cortex it captures approximately equal amounts of gyral and sulcal cortex, whether the central vertex is gyral or sulcal (Wagstyl et al., 2016). The code used is available from https://github.com/kwagstyl/FCDdetection/.

### 2.8 Normalisation of features

The following features were smoothed using a 10 mm FWHM Gaussian kernel - cortical thickness, grey-white matter intensity contrast and FLAIR signal intensity. In every individual, these features underwent two normalisation procedures. 1) Features were normalised using a within-subject z-scoring, that adjusts for inter-individual differences in the mean and standard deviation. 2) Features were normalised using a between-subject z-scoring, where each participant's per vertex feature was normalised by the mean and standard deviation in the population of healthy controls. This adjusts for inter-regional differences in the mean and standard deviation.

### 2.9 Interhemispheric asymmetry

Cortical thickness, grey-white matter intensity contrast, local cortical deformation and FLAIR intensity samples, were registered to an average space that has an identical number of vertices for each hemisphere (Greve et al., 2013). The right hemisphere vertex values for each feature were subtracted from the left hemisphere values to create a left hemisphere asymmetry map and visa versa for the right hemisphere. In the resulting asymmetry maps for each hemisphere, positive values indicated greater ipsilateral feature values while negative indicate that the contralateral hemisphere has a higher value for that vertex.

### 2.10 Statistical analysis

#### 2.10.1 Machine learning classification

The Neural Network Toolbox in MATLAB R2014a (The MathWorks, Natick, MA, U.S.A.) was used to create a nonlinear classifier. An artificial neural network is a group of interconnected nodes, each of which represents an artificial neuron. It is a supervised, feedforward network that can be trained to recognise complex patterns. This network has one-way connections from input to output layers and via a layer of hidden nodes. Each node is activated by a differently weighted combination of features, which are optimised during the training phase. The outputs of the hidden nodes are then combined to determine whether the set of features of that a particular vertex resemble healthy (output value closer to zero) or lesional (closer to one) cortex.

A single hidden layer neural network was chosen as the classifier as they can be rapidly trained on large datasets, are flexible and incorporate the capabilities of support vector machines. Unless otherwise stated, the number of nodes in the network was determined through running a principal component analysis (PCA) on the input surface-based features in the control cohort, and using the number of components that explained over 99% of the variance.

**Fig. 3:**
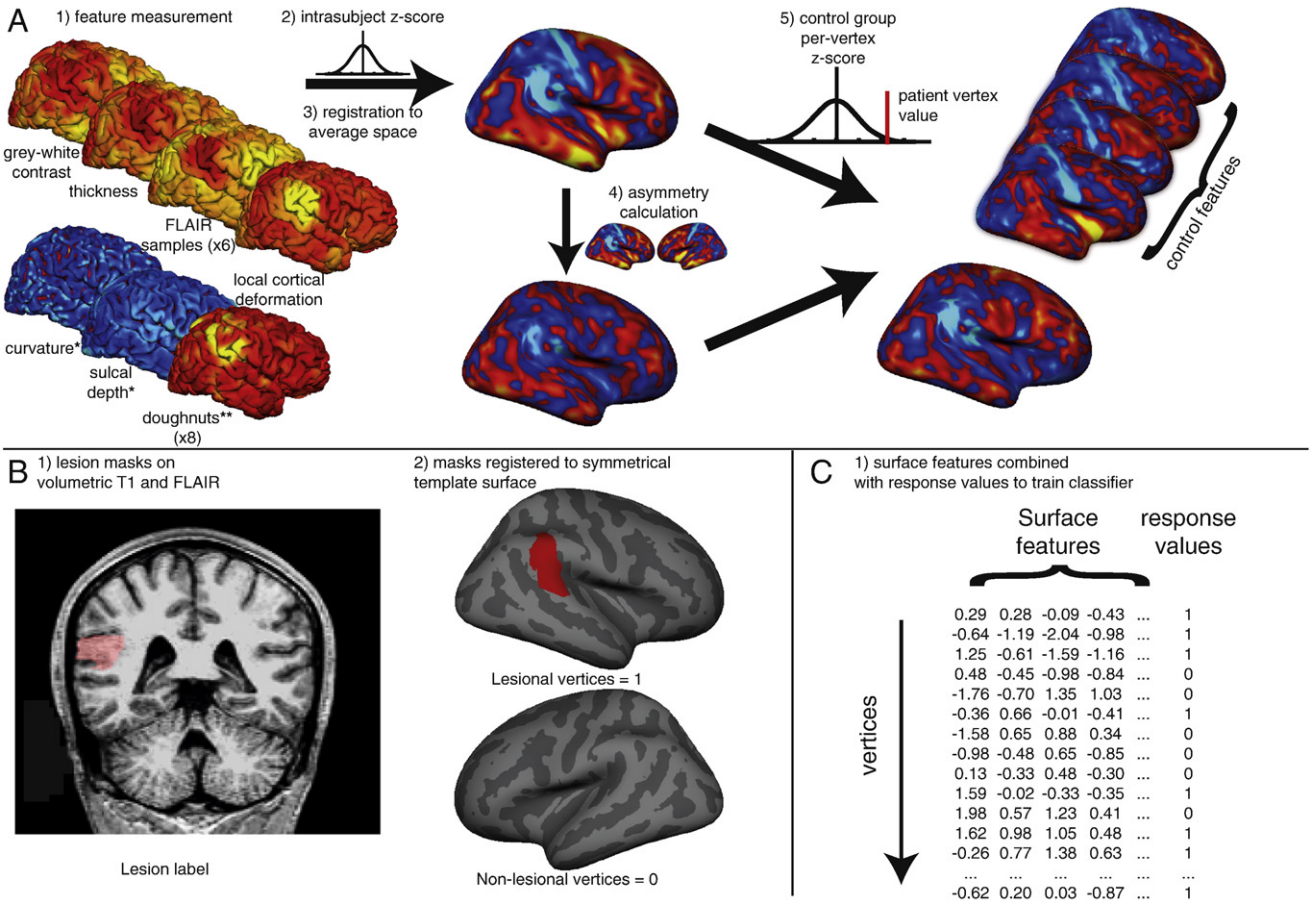
Overview of classifier. A) 1. Quantification of surface based features on each individual including established features – cortical thickness, FLAIR intensity (sampled at 6 cortical depths), grey-white contrast, curvature, sulcal depth – and novel features – “doughnut” method (for 6 FLAIR intensity samples, cortical thickness and grey-white contrast) and local cortical deformation (LCD). 2. Intra-subject normalisation (z-score). 3. Registration to the symmetrical template brain. 4. Per-vertex interhemispheric asymmetry calculations for each feature map. These serve to filter symmetrically extreme values such as thin primary sensory cortices. 5. Per-vertex normalisation by the controls of z-scored feature maps and asymmetry maps. These serve to filter common regional differences or asymmetries such as the planum temporale. * = feature undergoes steps 1, 2 and 5 only. ** = feature undergoes steps 1 and 2 only. B) 1. Volumetric lesion masks are manually segmented using T1 and FLAIR images. 2. Lesion masks are mapped to the surfaces and then to the symmetrical template brain. Lesional vertices are given a response value of 1, and contralateral non-lesional vertices are given a value of 0. C) 1. Neural network classifier is trained on surface based features and response values using leave one out cross-validation. Each row corresponds to a single vertex on one patient, each column to a surface based feature or the response variables.

Neural network classifiers were trained using surface based measures from vertices from each patient (Fig. 3). For the full network the 28 input measures were - normalised cortical thickness, normalised grey-white matter intensity contrast, sulcal depth, mean curvature, the 6 normalised FLAIR intensity samples at different cortical depths, normalised LCD, “doughnut” thickness, “doughnut” intensity contrast, “doughnut” FLAIR intensity at different cortical depths as well as the normalised interhemispheric asymmetry measures of cortical thickness, grey-white matter intensity contrast, the FLAIR intensity samples and local cortical deformation. Separate neural networks were also trained using individual surface based features and subsets of the full data to evaluate the discriminatory value of specific features. For those trained on individual features 2 nodes were included in the hidden layer to enable sensitivity to both abnormally high and low values.

Each vertex in the training dataset was given one of two response values — lesional cortex or healthy cortex. Vertices from within each lesion mask were given a response value of one, while vertices from contralateral hemisphere of each patient were given the value zero, i.e. healthy cortex. Ipsilateral healthy data was disregarded from the training set to minimise the number of misclassified vertices; for example where lesions extend beyond what is visible through conventional radiological analysis. Each classifier was assessed using a leave-one-out strategy, i.e. the neural network would be trained using data from 21 patients and then tested on the 22nd patient. The testing phase of the classifier outputs a probability map, where values closer to 0 are more likely to be healthy cortex and values closer to 1 are more likely to be lesional cortex.

The full matrix of data input to the neural network and reference list of features is available from the University of Cambridge's online data repository.

#### 2.10.2 Clustering

The output probability maps from the classifier are thresholded so that only the top 5% of vertices remain and surviving vertices are grouped into neighbour-connected clusters. The smallest clusters, below 200 vertices (~ 1 cm^2^) were excluded as noise. The cluster with the highest mean probability value is considered the putative lesion location. The automated lesion detection method is considered successful if this cluster overlaps the lesion mask. This final step is designed to always output one putative lesion location per test subject, as a radiological aid to FCD diagnosis. As a consequence, specificity cannot be calculated.

#### 2.10.3 Evaluation of novel features

Surface based features were evaluated using two methods - receiver operator characteristics of individual surface-based features and sensitivity of classifiers containing combinations of features.

To assess the discriminatory value of individual surface-based features receiver operator characteristics (ROC) and area under the curve (AUC) were calculated per vertex for the classifiers trained on each individual feature.

Evaluation of the full impact of these novel features was carried out by comparing the sensitivity of the classifier including novel features, to that of a classifier based on solely established surface-based features for FCD detection (normalised cortical thickness, normalised grey-white matter intensity contrast, sulcal depth, mean curvature and the 6 normalised FLAIR intensity samples). To evaluate whether local cortical deformation is a more sensitive marker of cortical folding complexity than local gyrification index, a subsequent analysis compared the sensitivity of the classifier with all novel features (including local cortical deformation) to the sensitivity of a classifier replacing local cortical deformation with local gyrification index.

#### 2.10.4 Assessment of demographics and movement artefact

Demographic variables between patients and controls, and motion ratings of FLAIR scans between detected and non-detected groups were compared using a Mann-Whitney *U* test in SPSS version 22.

## 3 Results

### 3.1 Demographics

A total of 22 patients with a radiological diagnosis of FCD and 28 healthy controls were included. Demographic information for the patients is available in Table 1. The sex of the control group was not significantly different to the FCD group (Mann-Whitney U: 431.50, p = 0.964). However, the median age of the control group did differ significantly from the FCD group (Mann-Whitney U: 251.00, p = 0.005). Based on the radiological reports the seizure focus was left-sided in 10 patients, right-sided in 11 patients and bilateral in 1 patient. Lesion location was largely split between involving the temporal lobe (N = 9) and the frontal lobe (N = 8), with only 2 lesions in the parietal cortex, 1 in the occipital lobe and 2 multi-lobar. Median subjective motion rating across all patients' FLAIR scans was 3 (range 1–5). At the time of study, 11 out of 22 patients with a radiological diagnosis of FCD had undergone focal resections. Seven resections met a histopathological diagnosis of FCD Type IIB, one FCD Type IIA, two demonstrated focal neocortical gliosis only and one did not in fact have an FCD, but a focal ganglioglioma (WHO Grade I) was evident from histological examination.

**Table 1:** Patient demographics.

No.	Age	Sex	Onset (yr)	Duration (yr)	MRI	Hemi	Current anti-convulsants	Motion/artefacts	Surgery	Histology	Engel	Detected
1	4.03	F	0.5	3.53	Peri-sylvian + temporal	R	LVT, VPA, CLB	4	y	Gliosis only	III	y
2	14.92	M	7	7.92	Inferior parietal	R	LVT, OXCBZ, CLB	3	y	FCD type IIB	III	y
3	14.93	M	8	6.93	Inferior temporal sulcus^⁎^	L	OXCBZ, LVT	2	y	FCD type IIB	Ia	y
4	9.06	M	2	7.06	Superior temporal sulcus	L	CBZ	3	y	FCD type IIB	Ia	y
5	14.31	F	2.5	11.81	Temporal pole	R	LVT, TPR	1	y	FCD type IIB	Ia	y
6	15.37	M	9	6.37	Middle temporal gyrus	R	CBZ, TPR	1	n			y
7	13.58	F	0.67	12.91	Medial occipital lobe^⁎^	R	LVT	2	n			y
8	9.04	M	1.92	7.12	Anterior temporal lobe	R	VPA, CBZ	3	y	Subpial gliosis	IV	y
9	3.79	M	3	0.79	Superior frontal sulcus	L	CLB, TPR, OXCBZ, PNT	3	y	FCD type IIB	Ia	y
10	13.51	M	4	9.51	Temporal pole	R	LTG, methylphenidate	5	n			n
11	15.2	M	10	5.2	Anterior temporal pole	L	OXCBZ, CLB	2	y	FCD type IIB	Ia	y
12	16.21	F	1	15.21	Mesial parietal^⁎⁎^	L	CBZ	5	n			n
13	14.77	F	6	8.77	Superior temporal gyrus	L	LVT, LTG	2	y	Ganglioglioma	Ia	y
14	14.21	F	0.67	13.54	Superior frontal sulcus	R	VPA, OXCBZ, perampanel	3	y	FCD type IIA	Ia	y
15	5.49	M	0.5	4.99	Temporal lobe	R	VPA, LVT, LTG, prednisolone	3	n			y
16	12.73	M	3	9.73	Middle frontal gyrus	L	OXCBZ, CLB	2	y	FCD type IIB	Ia	y
17	11.8	F	3	8.8	Temporal, occipital, posterior parietal lobes	L	LTG, CLB, OXCBZ	2	n			y
18	8.18	M	1.5	6.68	Lateral orbital gyrus^⁎^	R	VPA, CBZ	5	n			n
19	15.98	M	10	5.98	Precentral gyrus^⁎^	L	VPA, LVT, CBZ	3	n			n
20	15.58	F	5	10.58	Inferior frontal lobe	R	VPA, LVT	2	n			y
21	9.22	M	7	2.22	Superior frontal sulcus and precentral sulcus	R	VPA	3	n			n
22	13.82	F	11	2.82	Precentral sulcus^⁎^	L	CBZ	3	n			n

Age, onset, and duration are presented years; MRI: lesion location on MRI report, ^⁎^originally MRI -ve ^⁎⁎^participant had dental braces during MRI scan; current anti-convulsants: LVT = levetiracetam, CBZ = carbamazepine, OXCBZ = oxcarbazepine, VPA = sodium valproate, CLB = clobazam, LTG = lamotrigine, TPR = topiramate, PNT = phenytoin; Motion/Artefacts: ranking according to motion classification system; Engel: post-operative surgical outcome according to Engel classification (Engel, 1993), Ia = completely seizure free, III = worthwhile improvement, IV = no worthwhile improvement; Detected: y = classifier detects lesion as primary cluster, n = lesion undetected.

### 3.2 Assessment of novel surface-based feature maps

Qualitative analysis of the “doughnut” maps indicated that they may provide useful surface features for the detection of FCDs. By quantifying local changes in cortical thickness, grey-white matter intensity and FLAIR intensity (Fig. 1C) this method highlighted locally abnormal areas of cortex. However, these metrics were judged to be sensitive but relatively unspecific. For example, in small lesions, the centre of the lesion was often identified, whereas in larger lesions, it is the lesion boundaries that were detected. As well as identifying the lesion as an area of high variability in cortical structure, the “doughnut” method did identify many other areas of high cortical variability, thus suggesting their limited use in univariate analyses and the need for their use in combination with other features. Visual inspection of the “local cortical deformation” measure (Fig. 2) indicated that this metric was sensitive to abnormal lesion morphology. Interhemispheric asymmetry measures (Fig. 3) were of particular use in preventing normal anatomical variants from being considered abnormal. For example, the primary somatosensory cortex is normally very thin. As it is thin bilaterally, although it falls in the extreme values for cortical thickness, the interhemispheric asymmetry values for this gyrus were around zero. In contrast, the lesions were unilateral and therefore had abnormal unilateral cortical thickness values and abnormal asymmetry values. Overall, qualitative assessments of detection rate of lesions using the novel, surface-based features supported their incorporation into multivariate paradigms for lesion detection.

**Fig. 4:**
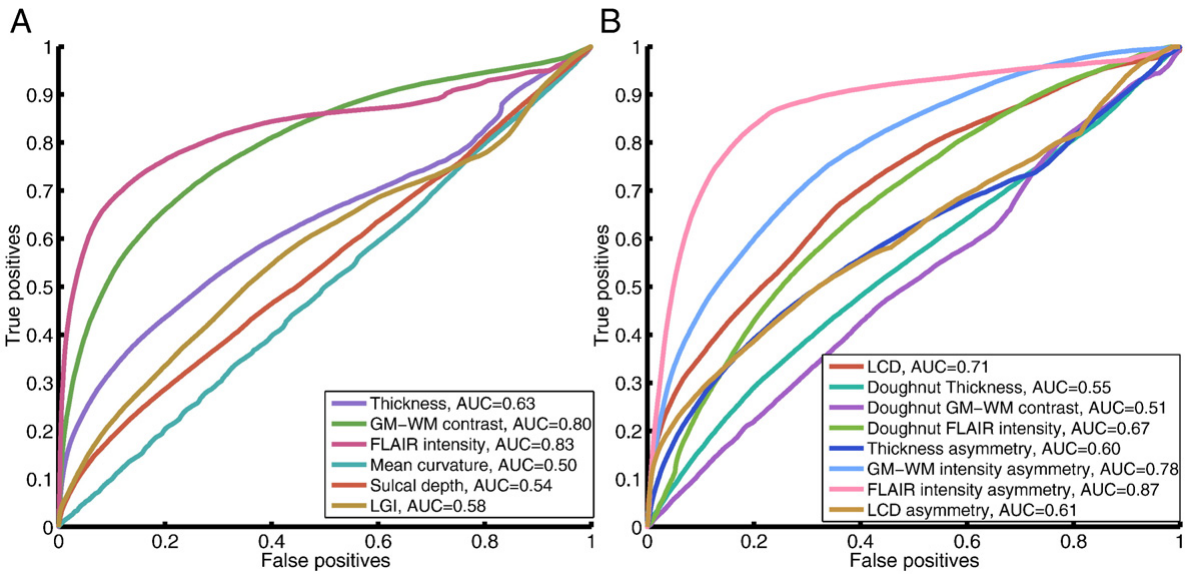
Receiver operator characteristics and AUC for classifiers trained on individual established (A) and novel (B) features. Within established features, FLAIR signal intensity, cortical thickness and grey-white matter intensity contrast are most discriminatory of lesional vertices. Within novel features, interhemispheric FLAIR intensity asymmetry, grey-white matter contrast asymmetry and local cortical deformation are the most discriminatory of lesional vertices.

For quantitative evaluation of individual established and novel features, receiver operating characteristics and area under the curve (AUC) were calculated using the output of a 2-node neural network classifier (to enable sensitivity for both abnormally high and low values) (Fig. 4). These revealed that individually, all novel surface-based features add some discriminatory value (AUC > 0.5). Of the established features, FLAIR intensity appeared most discriminatory (AUC = 0.83) followed by GM-WM contrast (AUC = 0.80) and thickness (AUC = 0.63). Individual novel features all added some discriminatory value (AUC > 0.5) with FLAIR intensity asymmetry performing highest across all measures (AUC = 0.87). It is important to note that these statistics were calculated on a per-vertex basis, and therefore do not differentiate between when all lesions are partially detected and when entire lesions are either detected or undetected by specific metrics. Moreover if there were any undiagnosed multifocal structural abnormalities outside of the radiological lesion mask, these would have appeared as false positives incorrectly reducing the AUC. Nevertheless, these results strongly suggested that classifiers might be improved by the incorporation of these novel features.

### 3.3 Establishing the parameters for the classifier

The principal component analysis using both novel and established features (No. of features = 28) in the control cohort revealed 11 principal components were required to explain over 99% of the variance compared to 6 when using solely established features (No. of features = 11). The neural network was therefore trained using the full 28 established and novel features with 11 nodes and 1 hidden layer. The sensitivity of the output of this classifier was then compared with classifiers trained using only the 11 previously established surface features. Two classifiers were trained and tested, one with 6 nodes and the other with 11 nodes - to prevent systematic bias introduced by differing neural network parameters.

### 3.4 Classification including novel features vs. classification using established features

**Fig. 5:**
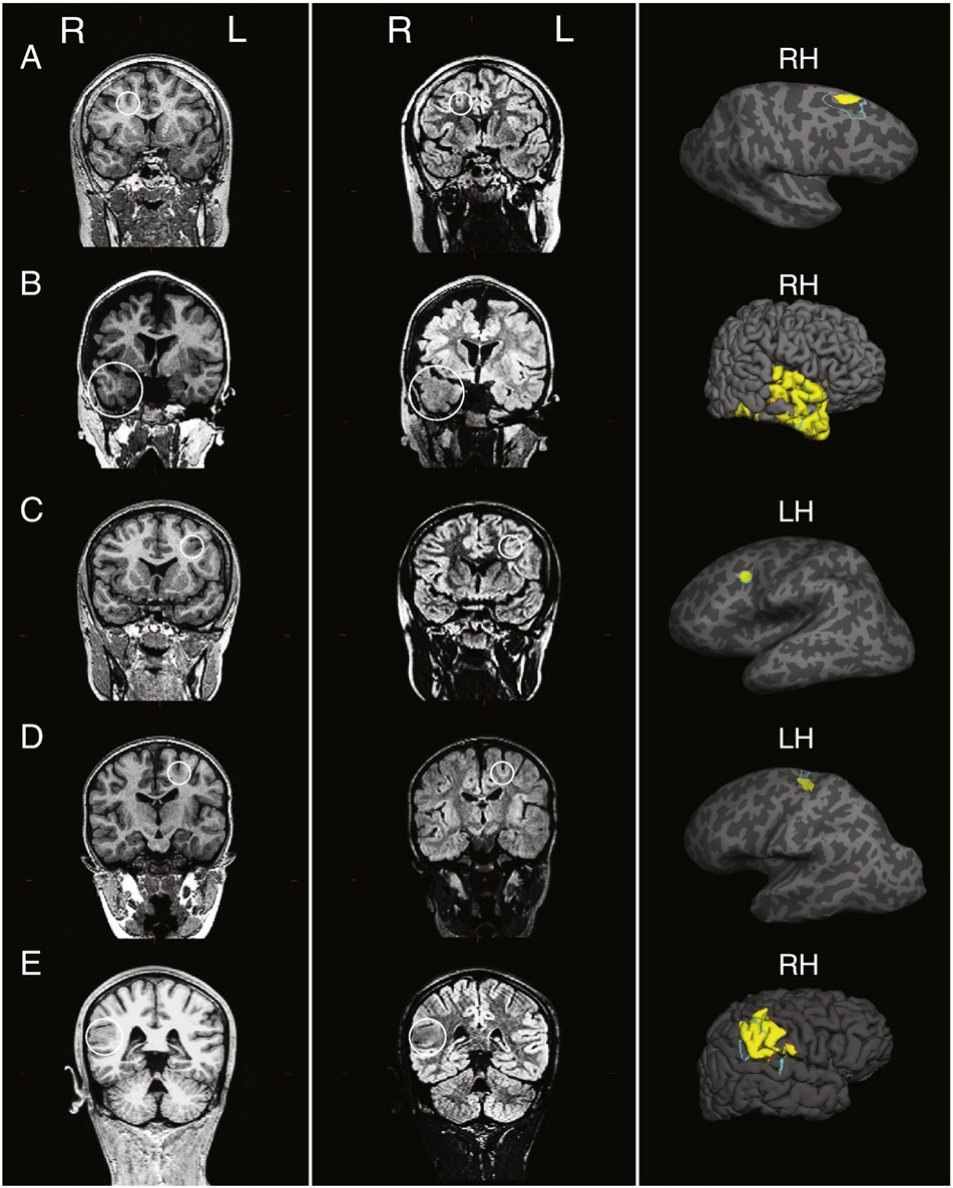
Examples of top cluster output in 5 patients with a radiological diagnosis of FCD. First column: T1-weighted images. Second column: FLAIR images. White circle on T1 and FLAIR images indicates lesion location. Third column: Top cluster of neural network classifier output (yellow) and manual lesion mask (light blue) viewed on pial surface, for large lesions, or inflated surface, for small lesions buried in sulci. For corresponding patient numbers in demographics table: A = 14, B = 15, C = 16, D = 9, E = 2.

The neural network involving novel and established features was able to detect 16 out of 22 FCDs (73%) as the putative lesion location (Fig. 5). Out of the remaining 6 cases, the lesion in one patient was detected as the 5th cluster, and in 5 patients their lesions were not detected as one of the top 5 clusters. Further inspection of the scans of the 5 undetected patients revealed large motion artefacts particularly on the FLAIR images. The median anonymised motion rating of FLAIR images was 3 for the undetected patients in comparison to 2 in the detected patients (Mann-Whitney U: 12, p < 0.019), which may account for why they were missed. There was no significant age difference between detected and undetected patients (Mann-Whitney U: − 0.48, p = 0.63).

In comparison, the neural network using only previously established surface features and 11 hidden nodes was only able to detect 12 out of 22 FCDs (55%) as the primary cluster, whilst with 6 hidden nodes (as established through a principal components analysis) was able to detect 13 out of 22 FCDs (59%), further evidence that inclusion of the novel features aided the detection of FCDs.

### 3.5 Local cortical deformation vs local gyrification index

**Fig. 6:**
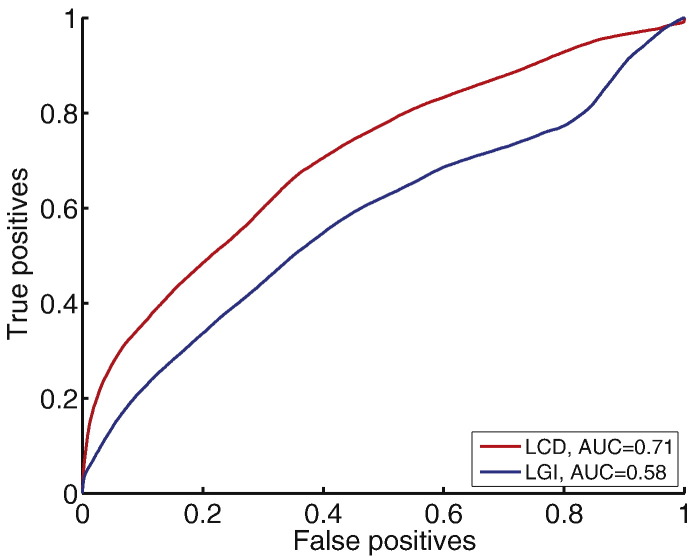
Receiver-operating characteristics and AUC comparing classifiers trained on local cortical deformation and local gyrification index. Of the two measures of cortical folding, local cortical deformation is superior to local gyrification index in discriminating lesional vertices from non-lesional vertices.

Measures of cortical shape, LCD and LGI, were directly compared both in terms of their individual discriminatory value and as inputs in the multivariate framework. In the AUC analysis of networks trained on a single feature, LCD (AUC = 0.71) performed much better than LGI (AUC = 0.58) (Fig. 6). In the full classifier containing 28 features, including LGI instead of LCD (11 nodes), the neural network was only able to detect 12 out of 22 FCDs (55%) as the primary cluster, significantly lower than the sensitivity when including LCD (73%).

## 4 Discussion

The automated FCD algorithm proposed here successfully identified FCDs despite the challenges of a paediatric population. Between ages of one and 18 there are large-scale structural changes to the cerebral cortex (Giedd et al., 2015, Gogtay et al., 2004, Raznahan et al., 2011, Shaw et al., 2008, Whitaker et al., 2016a, Whitaker et al., 2016b) including measurable changes to folding, thickness and myelination. By identifying local changes (both by looking at local changes using the “doughnut” method, as well as utilising abnormal structural asymmetries), implementing subtle morphological markers (LCD) and normalising within each subject, between hemispheres and with a paediatric control group, we demonstrated that it is possible to detect FCDs with a sensitivity of 73%. Previous studies have demonstrated that surface-based structural MRI, coupled with automated computational techniques can detect FCDs in adult cohorts (Ahmed et al., 2015, Hong et al., 2014). This therefore represents a potentially significant advance in the treatment of paediatric epilepsy.

The “doughnut” method introduced here was able to identify abnormal changes in cortical thickness, grey-white matter boundary intensity and FLAIR signal intensity across the cortex. Focal cortical dysplasias are often characterised by abnormal cortical thickness, grey-white matter boundary intensity and FLAIR signal intensity. However, there are normal changes in cortical structure that might obscure these changes. For example sulci are thinner than gyri (Brodmann, 1909, Economo Von and Koskinas, 1925) and small FCDs, characterised by cortical thickening, are often located at the bottom of sulci (Besson et al., 2008). Thus, a lesion at the bottom of a sulcus, may be abnormally thick relative to the surrounding sulcal cortex and yet a measurement of absolute thickness might still fall within the normal range for the cortex. However the “doughnut” method was specifically designed to obviate such difficulties by taking into account values of neighbouring vertices, to measure local changes. As such this method was sensitive to such subtle structural changes, that might be missed by solely considering values from isolated vertices, which has to date been the established approach (Ahmed et al., 2015, Hong et al., 2014). Importantly these “doughnut” maps could be calculated on any surface registered maps and could therefore be used to highlight local structural changes using a much wider range of measures or imaging modalities.

Local cortical deformation (LCD) maps small-scale alterations in cortical shape. For example, it would differentiate a golf ball from a smooth sphere by being sensitive to the dimples in the golf ball's surface. As it is based on intrinsic curvature, a mm-scale metric of cortical deformation, it is more sensitive to subtle shape abnormalities than LGI, a cm-scale measure (Ronan et al., 2011, Schaer et al., 2008). This was clearly demonstrated both through the increased AUC of a classifier trained on LCD compared to LGI, as well as the increased sensitivity of a classifier trained on the established and novel features that incorporated LCD, as opposed to LGI. It may therefore help to identify subtle shape changes in a wider range of disorders of cortical development (Ronan et al., 2012, Wagstyl et al., 2016).

Comparison of the ipsi- and contra-lesional hemispheres is an integral component of the radiological assessment of MRI scans. Interhemispheric registration of feature maps allowed for quantification of interhemispheric asymmetry of surface-based metrics at each vertex. This served to filter healthy but symmetrical interregional variations, such as bilaterally thin and heavily myelinated primary sensory cortices (Wagstyl et al., 2015) or regions showing differential but symmetrical developmental trajectories (Shaw et al., 2008). Importantly, commonly occurring interhemispheric asymmetries, such as the planum temporale (Geschwind and Levitsky, 1968), were subsequently filtered by normalising these asymmetry values with the control dataset. This approach for interhemispheric registration has obvious applications for detection of any unilateral abnormalities including other malformations of cortical development, strokes and tumours.

This study advances automated FCD lesion characterization and detection in a number of respects. First, previous work in adults has made use of voxel-based (Bernasconi et al., 2001, Colliot et al., 2006, Focke et al., 2008, House et al., 2013, Huppertz et al., 2005, Wagner et al., 2011, Wang et al., 2015) and surface-based structural features (Ahmed et al., 2015, Hong et al., 2014, Thesen et al., 2011) to which LCD could be a valuable addition particularly given its increased discriminatory value over the existing measure of shape, LGI. Second, combining features into multivariate classifiers has only been applied in adults (Ahmed et al., 2015, Hong et al., 2014). Data post-processing methods including the “doughnut” method, interhemispheric asymmetry and intra- and inter- subject normalisation were included to address specific problems in the paediatric population but may well aid lesion detection in adults. Third, studies in children have focused on voxel-based techniques (Riney et al., 2012, Wilke et al., 2014), where individual maps can be sensitive but are less readily combined into a multivariate classification tool. Thus, novel structural features, data post-processing methods and incorporation of surface-based features into a neural network classifier furthers the detection of FCDs in epilepsy.

Although we report a lesion detection sensitivity of 73% in our cohort, certain challenges were unavoidable – excessive head motion is a recognised problem causing artefacts (Ducharme et al., 2015), dental braces creating large artefacts, difficulty recruiting age matched controls for patients as young as 4 years old and 1.5T data. Indeed the undetected lesions demonstrated relatively increased motion artefacts. We deliberately used 1.5T data and included imperfect images, making the methods likely to be effective on routine clinical data from any centre/MRI scanner. One final limitation, which is generally applicable to FCD detection studies, is the issue of multifocal lesions (Fauser et al., 2009). In some patients, multiple FCDs can be identified either presurgically, histologically or in post-surgical assessment when a patient continues to have seizures. However in this study radiological assessment of each patient only identified single lesions and patients had undergone resection of a single epileptogenic zone, thus we designed the classifier to identify only the single most likely FCD in a given subject. When investigating multifocal lesions in the form of extra-primary clusters, categorizing abnormal tissue from image artefact without a gold standard method for their identification, remains a challenge. However, if multi-focal lesions were heavily hypothesized within an individual, the classifier could be adapted to identify a set of abnormal clusters. Despite these challenges automated lesion detection in children was achievable. The novel surface measures developed here demonstrated substantial improvements in lesion detection and in future studies, classifier performance is likely to be improved by the use of larger, better quality datasets.

## 5 Conclusions

Our work advocates development and incorporation of new surface-based measures for FCD detection, as well as re-emphasising the use of established surface-based measures and machine learning paradigms. These tools could be more generally applied in the detection of localized lesions such as polymicrogyria, gangliogliomas or dysembryoplastic neuroepithelial tumours (DNETs). Furthermore, improving the detection of FCDs in a paediatric cohort may assist in the selection, referral and subsequent pre-surgical evaluation of children with drug-resistant focal epilepsy by providing putative lesion locations that may aid conventional visual analysis by neuro-radiologists. Paediatric automated lesion detection, when considered alongside a patient's detailed medical history and examination, video-EEG, MEG, PET/SPECT and neuropsychological evaluation in multi-disciplinary team meetings, might enable earlier and more effective assessment for surgical intervention. For an individual, this could mean shorter duration of uncontrolled epilepsy, reduced anti-epileptic medication and their associated side effects and improvement in their cognitive outcome.

## Funding

This research was supported by the National Institute for Health Research Biomedical Research Centre at Great Ormond Street Hospital for Children NHS Foundation Trust and University College London. SA received funding from the Rosetrees Trust (A711). KW received funding from the James Baird Fund and the Wellcome Trust (WT095692MA). TB from Great Ormond Street Hospital Children's Charity (V1213 and V2416). LR and PCF are funded by the Wellcome Trust and the Bernard Wolfe Health Neuroscience Fund.
